# Advances in Nanomaterials for Drug Delivery

**DOI:** 10.3390/biomedicines11020399

**Published:** 2023-01-29

**Authors:** Sérgio R. S. Veloso, Elisabete M. S. Castanheira

**Affiliations:** Physics Centre of Minho and Porto Universities (CF-UM-UP) and LaPMET Associate Laboratory, University of Minho, Campus de Gualtar, 4710-057 Braga, Portugal

In recent years, nanomedicine has provided several high-performance tools for overcoming biomedical challenges, resulting in numerous patents. Most notably, drug delivery nanosystems have allowed for surpassing drug stability and solubility limits, improved routes for drug administration, reduced dose-associated toxicity, and enhanced target specificity by exploring both active and passive delivery strategies.

Combinations of various nanomaterials have opened up possibilities for the development of smart multifunctional drug delivery systems, such as the synergistic use of both therapeutic and diagnostic agents (i.e., theranostic agents) in a single drug carrier. These advancements have enabled the spatial and temporal manipulation of drug delivery systems and, thus, on-demand drug release upon an external stimulus. This control over drug release remains a major challenge, moving towards multimodal and multifunctional strategies as a means to optimize efficiency and efficacy.

Hence, the Special Issue “Advances in Nanomaterials for Drug Delivery” was created to attract academic and scientific communities within this biomedical field to contribute their works concerning developments in drug delivery systems based on nanomaterials, with this Editorial being dedicated to summarizing and highlighting the findings of the published papers (three reviews and eight original research articles).

Literature reviews deepen the understanding of the pharmacokinetics and biodistribution of nanomaterials, as well as strategies to improve the targeting and delivery of drugs. Cai et al. [1] addressed in detail the physiological and pathological functions of macrophages, the interactions between nanoparticles and the immune system, and the recent advancements in organic and inorganic drug delivery systems, including strategies for the passive and active targeting of macrophages and the application of macrophage-targeting nanoparticles for the diagnosis and treatment of several diseases.

The review by Marin et al. [2] dealt with recent developments in greener routes for the sustainable synthesis of carbon nanomaterials, which require lower temperatures or use waste/by products as the carbon source, also addressing the progress on the application of these materials as drug delivery agents.

The challenging delivery of drugs with poor water solubility was reviewed by Teixeira et al. [3], emphasizing the use of liposomes and albumin-based nanosystems, two major classes of nanocarriers with formulations already approved by the U.S. Food and Drug Administration (FDA). The authors provided a detailed review on their preparation and advancements with both classes of materials in passive and active targeting for cancer therapy.

Among the original research articles, Cristiano et al. [4] demonstrated that the therapeutic activity of bergamot essential oil and ammonium glycyrrhizinate, naturally derive compounds with good anti-inflammatory properties, could be improved through coencapsulation in ultradeformable nanocarriers. The authors demonstrated that the codelivery improved the release profile kinetics of ammonium glycyrrhizinate and the deformability properties of the resulting nanosystem. Additionally, the efficacy of the nanosystem was confirmed through a topical cutaneous administration on human volunteers.

In the work by Quirós-Fallas et al. [5], the encapsulation of curcumin in hybrid lipid polymeric nanoparticles improved its antioxidant activity, compared to free curcumin, and displayed an important in vitro cytotoxic effect on gastric adenocarcinoma cell lines.

Another piece of published research on natural compound-based nanosystems was carried out by Khater et al. [6], reporting on the use of quercetin-loaded chitosan nanoparticles for the treatment of colitis. The nanosystem was found to alleviate the inflammation and oxidative stress using a dextran sulfate sodium (DSS)-induced colitis model.

Müller et al. [7] developed siRNA-loaded calcium phosphate nanoparticles decorated with either RGD or IgG (whether specific or not for CD69), and found that the design and surface decoration of the nanoparticles impacted the p65 nuclear factor-kappa B (NF-kB) protein expression in inflamed leucocytes and endothelial cells in vitro.

Articles on novel nanosystems with antimicrobial activity were also published. Alfei et al. [8] reported dendrimer nanoparticles loaded with 2-(4-bromo-3,5-diphenyl-pyrazol-1-yl)-ethanol (BBB4), displaying very low minimum inhibitory concentrations against several isolates of *S. aureus* and *S. epidermidis*. The same group also reported on the encapsulation of a pyrazole hydrochloride salt in a cationic copolymer, which was bactericidal against *Staphylococcus aureus*, *Escherichia coli* and *Pseudomonas aeruginosa* [9].

Regarding the nanosystems for cancer therapy, Cardoso et al. [10] developed multistimuli-responsive solid magnetoliposomes for the controlled delivery of doxorubicin. The PEGylation was confirmed to be an essential step in enhancing the long-term storage stability and stealth properties of delivery systems, as well as the optimal thermosensitivity for a controlled release under mild-hyperthermia conditions.

Finally, Villano et al. [11] proposed the use of molecularly imprinting polymers (MIPs) for the production of smart nanoparticles that modulate enzymes of the matrix metalloproteinase (MMPs) family to restore the correct balance between MMPs and their tissue inhibitors (TIMPs) to address the treatment of cardiovascular diseases.

We consider this Special Issue to bring together a wide variety of articles contributing to a fundamental understanding of the principles, fabrications, and applications of traditional or innovative nanosystems that demonstrate an advantage over current clinical formulations, progressing the state-of-the-art in nanomaterials for drug delivery. It has been our pleasure to edit this Special Issue, and we hope the papers are of interest to academic and scientific communities in the field of nanomaterials for drug delivery.

## References

[1] Cai D., Gao W., Li Z., Zhang Y., Xiao L., Xiao Y. (2022). Current Development of Nano-Drug Delivery to Target Macrophages. Biomedicines.

[2] Marin D., Marchesan S. (2022). Carbon Graphitization: Towards Greener Alternatives to Develop Nanomaterials for Targeted Drug Delivery. Biomedicines.

[3] Teixeira S., Carvalho M.A., Castanheira E.M.S. (2022). Functionalized Liposome and Albumin-Based Systems as Carriers for Poorly Water-Soluble Anticancer Drugs: An Updated Review. Biomedicines.

[4] Cristiano M.C., D’Avanzo N., Mancuso A., Tarsitano M., Barone A., Torella D., Paolino D., Fresta M. (2022). Ammonium Glycyrrhizinate and Bergamot Essential Oil Co-Loaded Ultradeformable Nanocarriers: An Effective Natural Nanomedicine for In Vivo Anti-Inflammatory Topical Therapies. Biomedicines.

[5] Quirós-Fallas M.I., Wilhelm-Romero K., Quesada-Mora S., Azofeifa-Cordero G., Vargas-Huertas L.F., Alvarado-Corella D., Mora-Román J.J., Vega-Baudrit J.R., Navarro-Hoyos M., Araya-Sibaja A.M. (2022). Curcumin Hybrid Lipid Polymeric Nanoparticles: Antioxidant Activity, Immune Cellular Response, and Cytotoxicity Evaluation. Biomedicines.

[6] Khater S.I., Lotfy M.M., Alandiyjany M.N., Alqahtani L.S., Zaglool A.W., Althobaiti F., Ismail T.A., Soliman M.M., Saad S., Ibrahim D. (2022). Therapeutic Potential of Quercetin Loaded Nanoparticles: Novel Insights in Alleviating Colitis in an Experimental DSS Induced Colitis Model. Biomedicines.

[7] Müller E.K., Białas N., Epple M., Hilger I. (2022). The Peptide/Antibody-Based Surface Decoration of Calcium Phosphate Nanoparticles Carrying SiRNA Influences the P65 NF-ΚB Protein Expression in Inflamed Cells In Vitro. Biomedicines.

[8] Alfei S., Brullo C., Caviglia D., Piatti G., Zorzoli A., Marimpietri D., Zuccari G., Schito A.M. (2021). Pyrazole-Based Water-Soluble Dendrimer Nanoparticles as a Potential New Agent against Staphylococci. Biomedicines.

[9] Schito A.M., Caviglia D., Brullo C., Zorzoli A., Marimpietri D., Alfei S. (2022). Enhanced Antibacterial Activity of a Cationic Macromolecule by Its Complexation with a Weakly Active Pyrazole Derivative. Biomedicines.

[10] Cardoso B.D., Cardoso V.F., Lanceros-Méndez S., Castanheira E.M.S. (2022). Solid Magnetoliposomes as Multi-Stimuli-Responsive Systems for Controlled Release of Doxorubicin: Assessment of Lipid Formulations. Biomedicines.

[11] Villano A., Barcaro G., Monti S., Barbani N., Rizzo A., Rossin D., Rastaldo R., Giachino C., Cristallini C. (2022). Molecularly Imprinted Nanoparticles towards MMP9 for Controlling Cardiac ECM after Myocardial Infarction: A Predictive Experimental-Computational Chemistry Investigation. Biomedicines.

